# Comment on Maga et al. The Effect of Selective Occlusal Adjustment on the Disclusion Time Reduction and Symmetry of Occlusal Contacts of the Own Dentition Using Digital Occlusion Analysis in Patients with Temporomandibular Disorders. *J. Clin. Med.* 2025, *14*, 7007

**DOI:** 10.3390/jcm15124827

**Published:** 2026-06-22

**Authors:** Robert B. Kerstein

**Affiliations:** Independent Researcher, Boston, MA 02072, USA; tmjdoc@ix.netcom.com

## 1. Background

The authors of The Effect of Selective Occlusal Adjustment on the Disclusion Time Reduction and Symmetry of Occlusal Contacts of the Own Dentition Using Digital Occlusion Analysis in Patients with Temporomandibular Disorders, J. Clin. Med. 2025;14:7007 https://doi.org/10.3390/jcm14197007 [1] should be commended for studying the ICAGD Coronoplasty and Disclusion Time Reduction [2,3,4,5,6,7,8,9,10,11,12,13,14,15,16,17,18,19,20]. By employing this non-subjective, time-based, computer-guided selective enameloplasty, the authors reported that they improved occlusal parameters in TMD patients [1]. Their findings further corroborate those of multiple prior ICAGD and DTR studies that have shown repeatedly that chronic muscular TMD symptoms were resolved without patients requiring splints, orthotics, TENS, jaw repositioning, Botox injections, or physical therapy [2,3,4,5,6,7,8,9,10,11,12,13,14,15,16,17,18,19,20,21].

**Figure 1:**
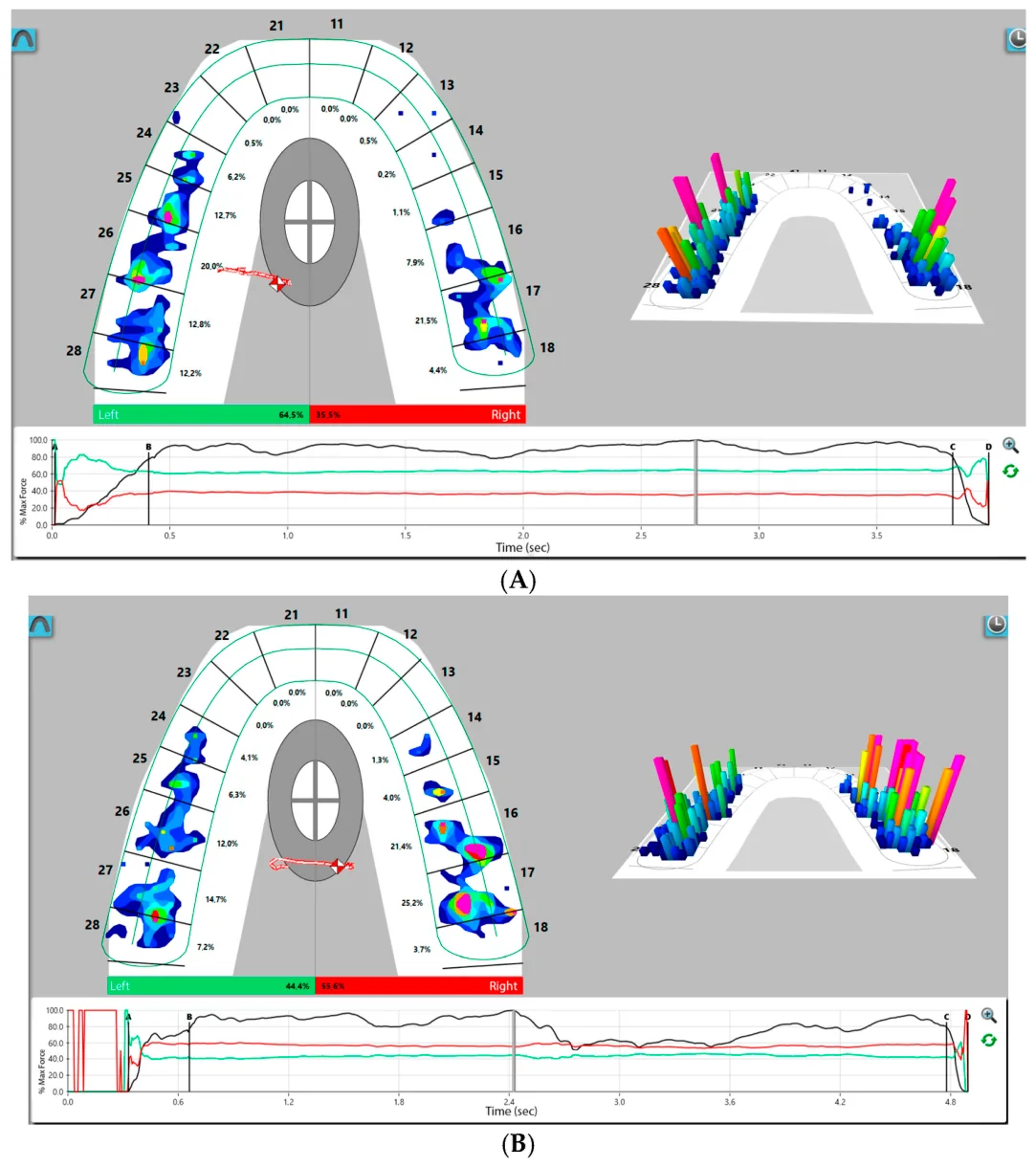
(**A**) has at least 6 pink saturated sensels, and (**B**) has at least 10 pink saturated sensels. Both figures have overestimated force percentages and report inaccurate occlusal force distributions because no sensitivity setting was performed prior to recording actual study data. A correct sensitivity level recording can be seen in Figure 2. Further, neither (**A**) or (**B**) Force vs. Time graphs exhibit an excursive recording. Both graphs show the subject closing into MIP from Lines A-B, holding intercuspation in MIP with variable closure strength between Lines B-C (the wobbling of the black total force line indicates the subject could not hold their MIP well), and then opening vertically out of MIP between Lines C-D. Correct excursive recording Force vs. Time graphs can be seen in Figure 3, Figures 6 and 7. The red and green lines represent the force % changes in the right (red) and left (green) arch halves. Their mostly horizontal character indicates the subjects were holding their MIP and not moving in an excursion.

With respect, despite the authors’ use of T-Scan 10 Novus technology (Tekscan, Inc., Norwood, MA, USA), and the fact that they calculated a number of measured occlusal function parameters (the Disclusion Time, the Occlusion Time, and the occlusal force distribution), within their presented T-Scan data figures (Figure 1A,B), there are visible T-Scan recording technique errors that compromised the reported Disclusion Times, the occlusal force distributions, and the COF positioning data. Most notably, and based upon their Methods, the authors did not set individual subject recording sensitivity levels before gathering study data. This means all T-Scan 10 high-definition recording sensors used (Novus HD, Tekscan Inc., Norwood, MA, USA) were not properly matched to each individual subject’s occlusal force strength range. This is always important to perform when recording patient function [17], but it was especially required in their specific study because subjects were “*instructed to bite down on the sensor with maximum force, following the initiation of recording via the control head*…”. By not setting the correct sensitivity per subject, sensors electronically overresponded to applied maximum forces, and then contact force levels were overreported, and falsely improved occlusal balance distributions were also reported. Figure 1A,B demonstrate excessively high numbers of pink saturated sensels, which clearly indicates that no pre-study data-gathering sensitivity setting took place.

**Figure 2:**
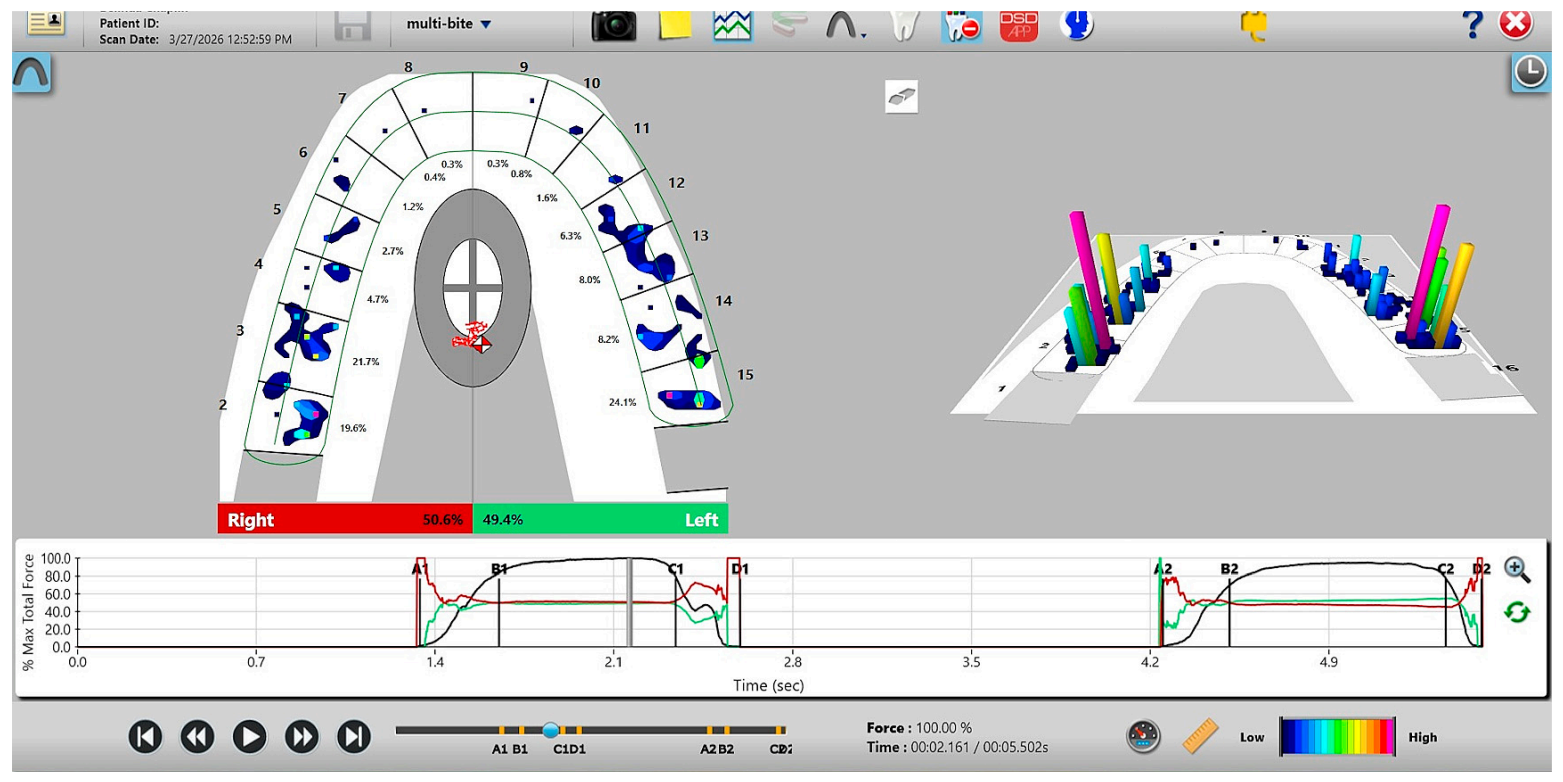
Limiting the sensor response to 1–3 pink sensels at maximum force (100%) minimizes sensor response and ensures 2197 sensels can properly grade the patient’s relative contact forces so that no occlusal parameter data is overestimated when subjected to statistical analysis. One can see actual optimal occlusal balance (left 48.8–right 51.2%) with far less pink saturated data compared to Figure 1B, where excessive sensor electrical response falsely balanced the occlusion.

**Figure 3:**
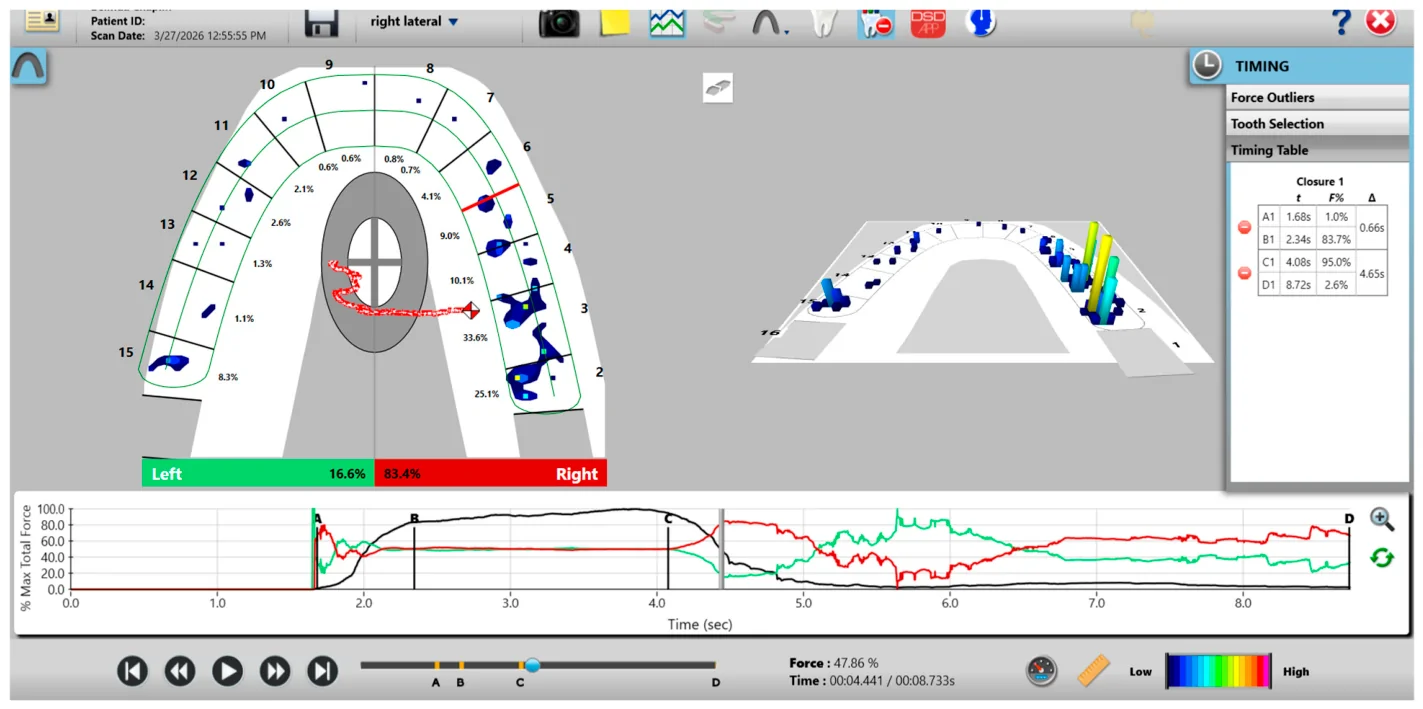
The C-D time duration is markedly extended within the Force vs. Time graph when excursive data is properly recorded. In this right excursion, the red (right arch half) and green (left arch half) force lines travel across the screen for significant time between C-D (the Disclusion Time = 4.65 s). When a patient moves laterally, over time posterior teeth transition across each other and contact forces lessen. Here the COF moves sideways into the 1st molar because an early group function controls this right excursive movement. Compared to Figure 1A,B (DT = 0.2–0.3 s) the C-D time period is significantly longer.

## 2. Understanding Why Sensitivity Setting Is Necessary

Proper sensitivity setting limits the T-Scan 10 HD sensor from electronically over-responding to occlusal surface contact [17]. Higher sensitivities input excess electrical charge into the sensor, which makes each of the 2200 recording sensels overly electronically responsive to occlusal compression. This directly results in many saturated (pink) sensels being distributed around the arch (as in Figure 1A,B). The T-Scan 10 software cannot count the amount of electricity (known as *Digital Output*) that resides in saturated sensels. Whereas setting the sensitivity to display only three saturated pink sensels when the patient compresses the sensor with maximum force ensures the other 2197 sensels’ Digital Output can be counted. Then the T-Scan 10 software can properly grade each sensel’s Digital Output count within 255 relative occlusal force levels using an 18 color-coded force scale (Figure 2 and Figure 3, bottom right color scale); very low force (dark blue; DO > 1 ≤ 42), low-medium force (blue; DO > 42 ≤ 85), medium force (green; DO > 85 ≤ 128), medium-high force (yellow-orange; DO > 128 ≤ 171), highest countable force (red; DO > 172 ≤ 255), and saturated pink (an unknown amount of high force DO > 255).

In Figure 1A, the occlusal force imbalance with six pink sensels (double the number of ideal sensel saturation) equaled left 64.5–right 35.5%. But in Figure 1B, with 10 pink sensels (triple the number of ideal sensel saturation), the balance improved to become left 44.45–right 55.6%. The authors likely believed the balance improved by adjusting the occlusion, but it was actually the result of excessive electricity running through the post-ICAGD sensor. Clinically, when T-Scan data is excessively pink, it leads operators to over-adjust certain contacts because falsely high force levels are being displayed. Setting correct sensitivity aids in controlling over-adjusting the occlusion [17].

Research-level and clinically correct T-Scan data to use when treating patients exhibits minimal pink saturation at maximum recorded force (Figure 2). This proper sensor electronic response does not falsely equalize occlusal balance (as in Figure 1B), does not magnify the force load percentages/tooth, and does not influence the Occlusion and Disclusion Time durations because the sensor surface does not respond when rubbed against enamel. The sensor only responds electronically when opposing occlusal surfaces very closely approximate each other. It is important for researchers to understand that validated recording techniques for both closure-into-MIP and lateral excursions have been defined and repeatedly tested since the early 1990s, being described in many T-Scan publications that predate this study by decades [3,5,12,13,17,18,19].

## 3. Incorrect Excursive Recordings

Despite that, this study attempted to record and measure excursive movement Disclusion Times, Figure 1A,B are not excursive recordings at all. They show the patient occluding into MIP, trying to hold their teeth intercuspated for 10 s, and then opening vertically out of MIP. The Force vs. Time graphs (bottom pane) are not representative of an excursive recording, but of a “closure only” recording. The C-D period is very short (0.2–0.3 s long), which is not representative of how an excursive T-Scan recording clinically appears. Nor did the authors report pre-ICAGD Disclusion Times (mean = 0.8 s) that reflect properly recorded excursive C-D time durations that have been reported in many prior ICAGD and DTR research publications [2,3,4,5,6,7,8,9,10,11,12,13,14,15,16,17,18,19,20,21].

The authors stated that “*after ICAGD, subjects were required to make lateral excursions while maintaining intercuspation on the sensor, continuing until posterior disclusion occurred*” [1]. But no T-Scan graphical evidence was presented that shows that research-level excursive recordings were made nor analyzed. Figure 1B legend states the recording was a post-ICAGD outcome, but it does not illustrate an excursive movement.

Note, in Figure 3 the C-D period where the Disclusion Time is correctly measured, is much wider “time wise” than in Figure 1A,B. The red and green force lines travel across the screen from Line C (excursive commencement) towards Line D (measurable complete posterior disclusion) for significant time, while the black total force line gradually drops from C towards the *x*-axis. These three lines are very different in form than the red, green and black graph lines in Figure 1A,B. Figure 3 shows that a correctly recorded lateral excursion requires much more time to evolve than when a patient simply vertically opens their teeth out of MIP.

## 4. Author Misstatement

Lastly, the authors made this complete misstatement; “the findings provide valuable *preliminary evidence on the clinical usefulness of ICAGD enameloplasty combined with digital occlusion analysis*, and highlight the need for further, long-term, randomized, and multicenter studies to validate and extend these observations” [1]. There are multiple ICAGD and DTR treatment studies, including randomized designs [4], that have permeated the literature over the past four decades (many more exist than have been cited in this *Comment*). These treatment studies performed in research environments illustrated that DTR and ICAGD have successfully resolved chronic occlusal maladies for which traditional unmeasured occlusal adjustments have found no therapeutic solutions [2,3,4,5,6,7,8,9,10,11,12,13,14,15,16,17,18,19,20,21].

“Preliminary” Disclusion Time Reduction and ICAGD evidence was first published in the 1990s using T-Scan I and II sensors and hardware electronics (Tekscan, Inc. S. Boston, MA, USA) [18,19,20]. During those years, the recording errors these authors made were clinically addressed and electronically resolved, as early sensor components evolved and T-Scan hardware became Windows-based (the T-Scan 10 Novus HD sensor is the 5th sensor generation). From there, reliable recording and data analysis procedures were clinically refined. As such, ICAGD and DTR are now many years beyond the *preliminary stage*.

Importantly, readers should know that in multiple patient treatment studies, ICAGD and Disclusion Time Reduction have been shown to:

rapidly decrease excursive masticatory muscle hyperactivity [3,4,8,13], mitigate pain [2,3,12], improve mastication strength, speed and motion patterns [6,7], reduce temporal headaches [2,4,5,12], reduce bruxism frequency and intensity [9,12], improve forward head posture [10], markedly reduce stress and salivary cortisol levels [16], improve emotional well-being [4,8,11,16] and lessen Trigeminal Neuralgia frequency and intensity [15], all without patients using occlusal appliances, undergoing jaw repositioning, Botox injections, transcutaneous electrical nerve stimulation (TENS), physical therapy, or using medication [2,3,4,5,6,7,8,9,10,11,12,13,14,15,16,17,18,19,20,21].

**Figure 4:**
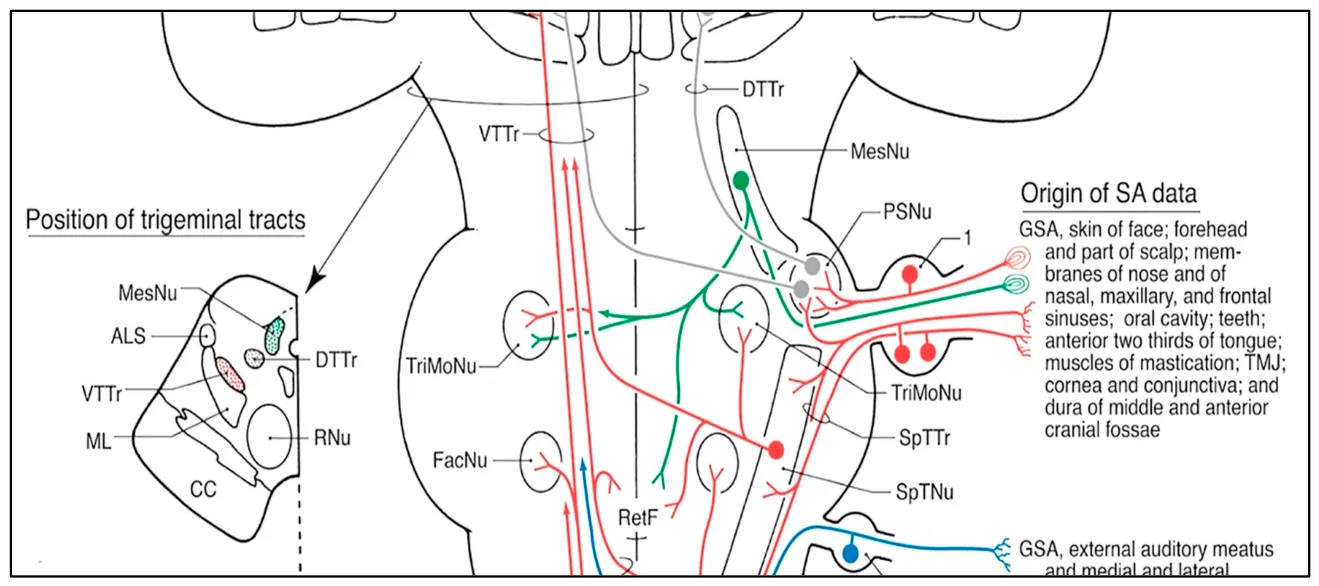
General somatic afferent nerve pathway (in green) from molar and premolar pulp and PDL mechanoreceptors that passes through the Mesencephalic Nucleus (MesNU) to first synapse with the Trigeminal Motor Nucleus (TrMoNU) and the Brain’s Reticular Formation (RetF). Reprinted from Kerstein R.B. Handbook of Research on Computerized Occlusal Analysis Technology Applications in Dental Medicine. IGI Global Scientific Publishing, 2015; p. 275 [23].

ICAGD and DTR physiologically relax the Swallowing Mechanism muscle group from within the central nervous system, by minimizing posterior tooth Periodontal Ligament compressions and pulpal flexures that directly synapse with the Trigeminal Motor Nucleus (Figure 4) [22].

## 5. Neurologic Pathway Between Shortening the Disclusion Time and Masticatory Muscle Activity Level Reductions

The molar and premolar pulp and Periodontal Ligament (PDL) mechanoreceptors are part of the Peripheral Nervous System (PNS) that lie outside of the brain and spinal cord, including the autonomic, cranial, and spinal nerves [24]. Peripheral nerves make their initial synapse outside of the Central Nervous System (CNS) before entering the spinal column, or the brain itself.

However, the molar and premolar pulp and Periodontal Ligament mechanoreceptors have unique neuroanatomy in that despite being peripheral nerves, they are the only afferent peripheral nerves that enter the CNS directly via the Mesencephalic Nucleus, and without synapsing there, travel on further to the Trigeminal Motor Nucleus bilaterally, while also traveling on to the Reticular Formation (Figure 4) [22]. The Reticular Formation is major brain center that controls swallowing, sleep, posture, respiration and digestion [25]. Because of this direct pathway from the posterior PDL and pulp mechanoreceptors to the Brain’s Reticular Formation, a 2026 DTR publication reported that 30 TMD participants undergoing ICAGD demonstrated a 50% reduction in mean salivary cortisol levels (from 11.43 ±1.48 ng/mL pre-ICAGD to 6.57 ± 1.20 ng/m post-ICAGD, =4.85 ng/mL reduction (*p* < 0.001). This is the first occlusal adjustment study in the literature to show a brain-mediated hormonal stress response was markedly lessened from computer-guided occlusal adjustments [16].

In Figure 4, the green pathway illustrates the pulpal and PDL mechanoreceptor afferent nerve pathway responsible for efferently elevating muscle activity when there are prolonged posterior excursive contacts (Disclusion Time ≥ 0.5 s/excursion). That same pathway can be neuro-electrically controlled via ICAGD time-based excursive adjustments that shorten contact durations down to known physiologic tolerances (Disclusion Time ≤ 0.4 s/excursion). This directly lessens the afferent electrical volume that leaves the posterior teeth and directly reaches the Trigeminal Motor Nucleus.

**Figure 5:**
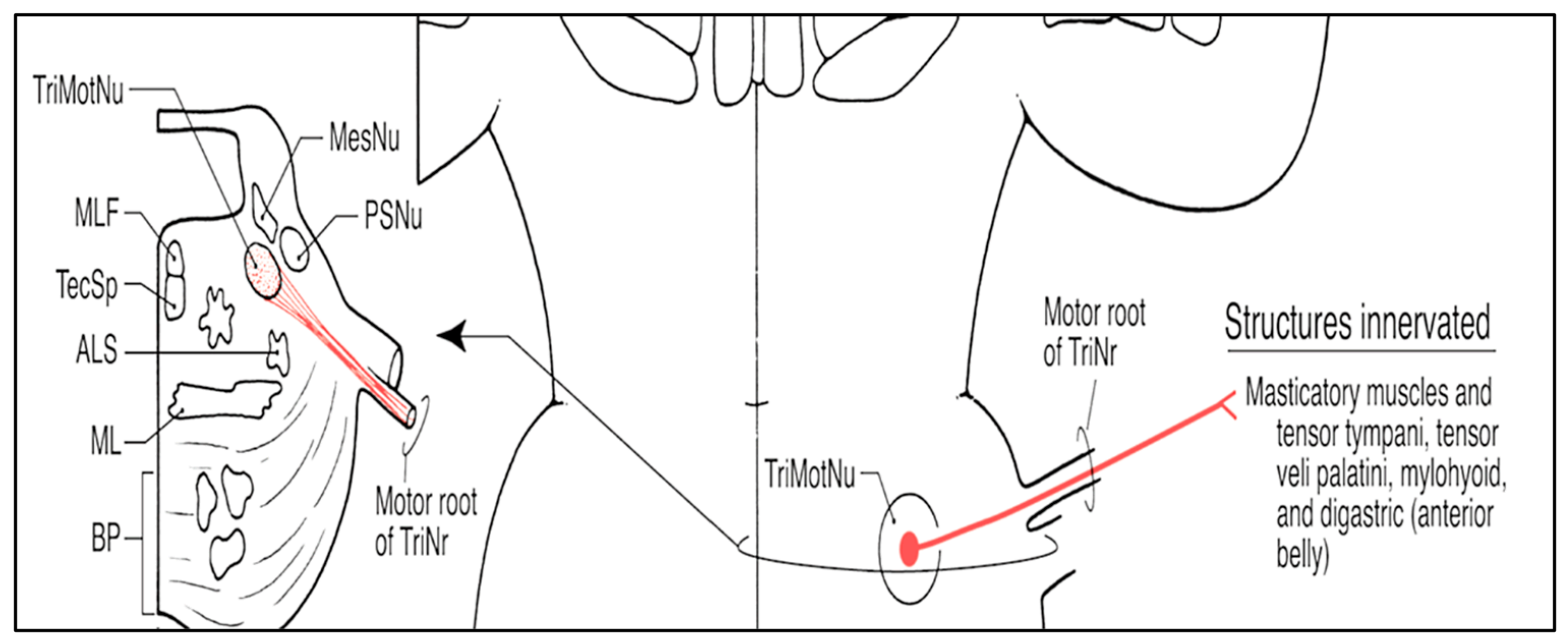
Structures innervated by the Trigeminal Motor Nucleus (TrMoNU). With prolonged posterior Disclusion Time, the TrMoNU efferently transmits the afferent high electrical input coming off the posterior teeth into high muscle firing within the Swallow Mechanism muscle group. This pathway contributes to the appearance of chronic muscular symptoms. Reprinted from Kerstein R.B. Handbook of Research on Computerized Occlusal Analysis Technology Applications in Dental Medicine. IGI Global Scientific Publishing, 2015; p. 276 [23].

Within the Trigeminal Motor Nucleus, the pulpal and PDL mechanoreceptor fibers make their first synapse with the efferent motor fibers to the four muscles of mastication: the tensor tympani, the tensor veli palatini, the mylohyoid, and the anterior belly of the digastric muscles, which comprise the Swallow Mechanism muscles (Figure 5). Creating very short Disclusion Time afferently lowers posterior tooth electrical output volume into the CNS, which directly lessens efferent electrical output volume leaving the Trigeminal Motor Nucleus to enter the swallow muscles. This, in turn, lowers muscle activity levels toward baseline when patients perform an excursive movement.

**Figure 6:**
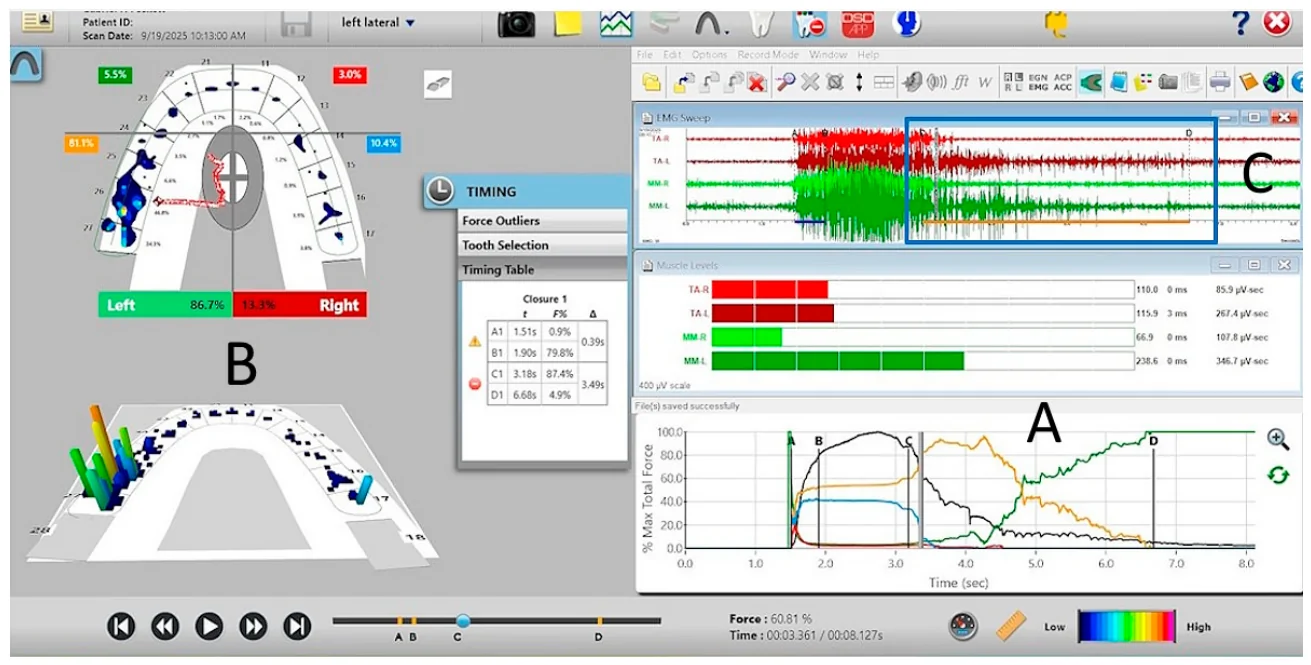
A pre-ICAGD T-Scan EMG left excursive recording where the excursion commences at Line C (**Force vs. Time graph,** (**A**)). In the T-Scan data (**left pane** (**B**)), the COF trajectory moves straight into the posterior left teeth as group function forces increase on the left side. In the **Force vs. Time graph** (**A**), the black total force line to the right of Line C has multiple small force drops that appear as “steps”, from opposing excursive contacts blocking free mandibular movement across posterior occlusal surfaces. The **Force vs. Time graph** (**A**) details that the orange posterior left quadrant force line completely controls the first half of the left excursion (posterior group function is in control). Mid-excursion (at 4.8 s), the left anterior green quadrant force line rises to 50% and begins to share the guidance with the posterior left orange quadrant force line. Later, as the orange quadrant line drops to 0% force, the left green anterior quadrant line reaches 100% force (at 6.68 s) marking the time-moment of complete posterior disclusion. Simultaneously in the **upper right EMG pane** (**C**) to the right of Line C continuing towards Line D **within the blue box**, there is extensive muscle contractility occurring in the left temporalis (TA-L), and both masseter muscles (MM-R, MM-L). The pre-ICAGD Disclusion Time = 3.49 s (**Timing Pane**). Reprinted with Open Access from Kerstein RB, Radke J, Sutter BA, Thumati P, Girouard P. Canine Protected Occlusion (CPO) Assessed from the Neurological and Muscle Activity Reducing Evidence-based Perspectives. *Adv Dent Tech*; 2026: 1–30 [21].

**Figure 7:**
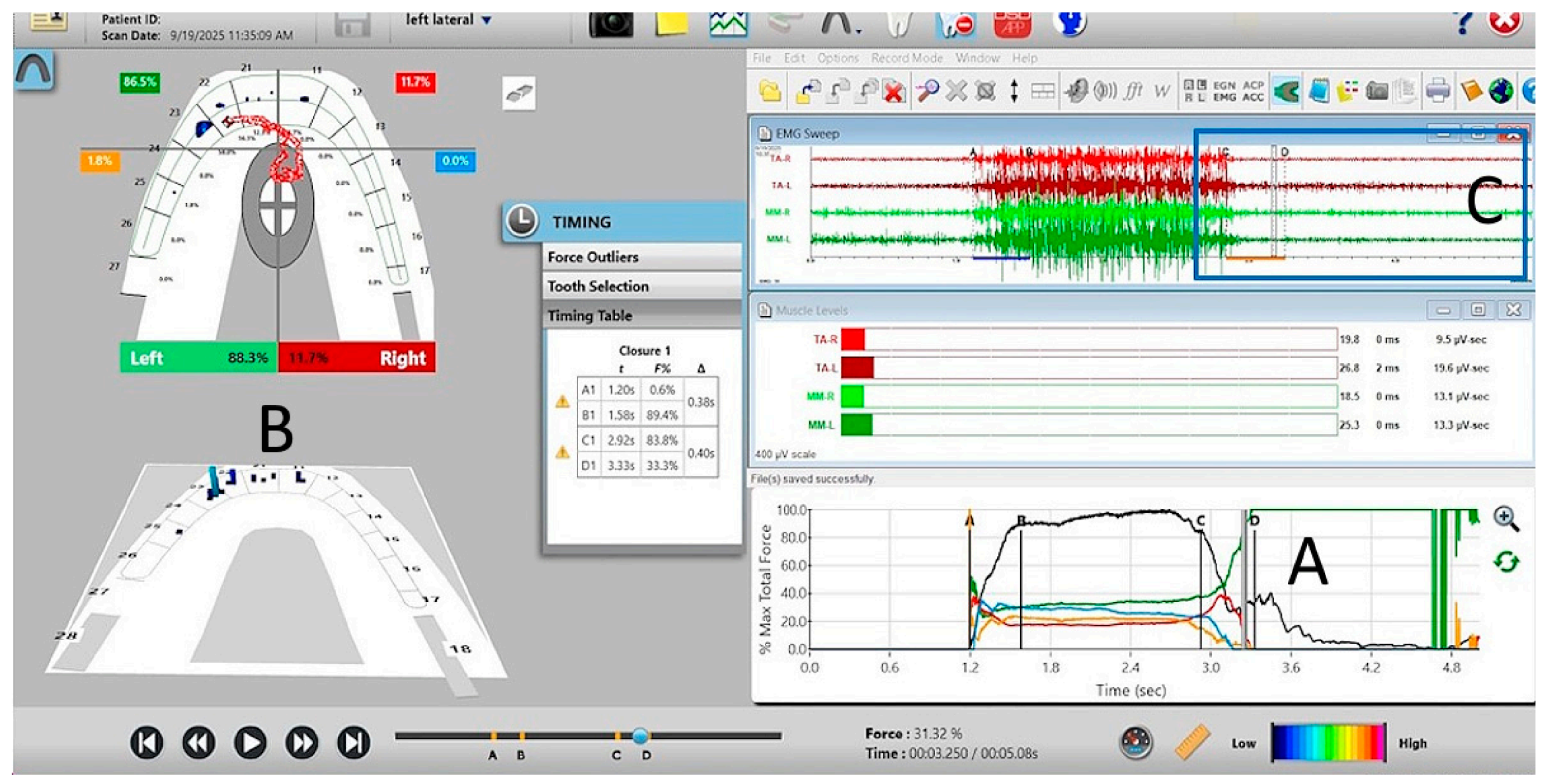
Post-ICAGD on the same day and in the same patient shown in Figure 6, to the right of Line C in the **Force vs. Time graph** (**A**), there is near-complete posterior disclusion of the left excursion (Line D) that occurred within the required timing duration tolerance (DT = 0.4 s; Timing Pane). In the **Force vs. Time graph** (**A**) just after Line C, the left anterior green quadrant force line quickly rises to 100% force as the posterior left quadrant orange and blue posterior right quadrant force lines quickly drop to 0% force. This Disclusion Time is 88.5% shorter than pre-ICAGD condition (Figure 6; DT = 3.49 s). After ICAGD, in the **T-Scan pane** (**B**), the COF moves towards the left canine, as all posterior teeth are discluded except for 1 premolar contact (tooth #25 palatal). Much less muscle activity is present to the right of Line C (**upper right EMG pane within the blue box** (**C**)) compared to the pre-ICAGD EMG data (Figure 6). Reprinted with Open Access from Kerstein RB, Radke J, Sutter BA, Thumati P, Girouard P. Canine Protected Occlusion (CPO) Assessed from the Neurological and Muscle Activity Reducing Evidence-based Perspectives. *Adv Dent Tech*; 2026: 1–30 [21].

Since 2004, when T-Scan 7 (Tekscan Inc., S. Boston, MA, USA) was first synchronized with electromyography hardware and software (BioEMG III, Bioresearch Assoc., Milwaukee, WI, USA), ICAGD is properly performed using simultaneous electromyomgraphic recordings of the masseter and temporalis muscles, which Maga et al., did not employ [26]. Importantly, research-level excursive movement T-Scan recordings that assess the Disclusion Time are best analyzed in quadrants. This separates the anterior teeth forces from the posterior teeth forces for better calculation of the time posterior teeth make excursive contacts [17]. T-Scan 10/BioEMG Figure 6 and Figure 7 illustrate the neurophysiologic muscle activity reductions that ICAGD produces when prolonged Disclusion Time (Figure 6) is treated to ≤0.4 s (Figure 7).

Significantly, adjusting the occlusion unmeasured with paper only and visual inspection of ink markings, or outside of the Disclusion Time ≤ 0.4 s/excursion range, means that markedly reduced muscle activity levels will not be predictably obtained, which then can maintain patient chronic symptoms resulting from higher-than-baseline excursive muscle activity levels. Essentially, the degree of muscular healing and the therapeutic success of the occlusal adjustments will be compromised. Whereas performing ICAGD properly and staying within the validated Disclusion Time numerical tolerances, a marked neurophysiologic muscle activity change will be therapeutically obtained (Figure 7) [2,3,4,5,6,7,8,9,10,11,12,13,14,15,16,17,18,19,20,21].

## 6. Reviewer Responsibility

Unfortunately, some of this incorrect data presentation and the non-questioning of the author’s opinion that this study provided “*preliminary evidence of DTR efficacy*” rests with the Journal itself. Had a trained, knowledgeable T-Scan reviewer read the original manuscript, they would have not only required more DTR and ICAGD literature be included in its’ Reference list, but also would have required significant corrections be made to Figure 1A,B, and to the manuscript itself before acceptance could be considered.

Owning a T-Scan in a university department or in a dental practice does not mean the owner understands its clinical use. T-Scan is an acquired skillset that involves (a) learning to record high-quality occlusal function data, (b) learning time and force-based data diagnosis from understanding how to employ the differing quadrant force and timing lines within the Force vs. Time graph, and (c) learning how to use that timing and force diagnosis to make targeted, high-precision, computer-guided, micro-adjustments [17]. Those three skills cannot be attained without a T-Scan dentist undergoing clinical chairside training with live patients. But all three learned skills begin with first learning research-level recording technique.

## 7. Recommendations

- In the future, it is very important that the Journal seek out a knowledgeable T-Scan reviewer to assess T-Scan research manuscripts for their correctness and accuracy before acceptance.
- T-Scan researchers trying to publish relevant T-Scan science should undertake clinical T-Scan training to learn proper T-Scan user skills prior to recording study data. That way, any reported occlusal function parameters will not be over or underestimated, and the recorded data will be of research-level quality, having been obtained with scientifically validated recording procedures [2,3,4,5,6,7,8,9,10,11,12,13,14,15,16,17,18,19,20].
- Dentists hoping to safely and effectively use the T-Scan 10 technology with patients should seek out proper T-Scan clinical user training. There are in-person live-patient clinical learning opportunities, as well as didactic courses available for any dentist who wants to learn how to therapeutically employ the T-Scan.

## Abbreviations

The following abbreviations are used in this manuscript:

ICAGD	Immediate Complete Anterior Guidance Development Coronoplasty
DTR	Disclusion Time Reduction Therapy

## References

[1] Maga W., Schönborn M., Pihut M. (2025). The Effect of Selective Occlusal Adjustment on the Disclusion Time Reduction and Symmetry of Occlusal Contacts of the Own Dentition Using Digital Occlusion Analysis in Patients with Temporomandibular Disorders. J. Clin. Med..

[2] Uchale P., Deogade S., Khalikar A., Wankhade S., Taneja S., Lalsare S. (2024). Effectiveness of T-Scan technology in identifying occlusal interferences and its role in the management of temporomandibular disorders: A systematic review. J. Clin. Diagn. Res..

[3] Kerstein R.B., Radke J. (2012). Masseter and temporalis excursive hyperactivity decreased by measured anterior guidance development. Cranio.

[4] Thumati P., Thumati R.P., Poovani S., Sattur A.P., Srinivas S., Kerstein R.B., Radke J. (2021). A multicenter disclusion time reduction (DTR) randomized controlled occlusal adjustment study using occlusal force and timing sensors synchronized with muscle physiology sensors. Sensors.

[5] Thumati P., Manwani R., Mahantshetty M. (2014). The effect of reduced disclusion time in the treatment of myofascial pain dysfunction syndrome using immediate complete anterior guidance development protocol monitored by digital analysis of occlusion. Cranio.

[6] Kerstein R.B., Radke J. (2017). Average chewing pattern improvements following disclusion time reduction. Cranio.

[7] Kerstein R.B., Radke J. (2019). Computer-guided occlusal treatment improves the smoothness timing and velocity of gum chewing. Adv. Dent. Technol. Tech..

[8] Thumati P., Poovani S., Ayinala M. (2023). A retrospective five-year survey on the treatment outcome of disclusion time reduction (DTR) therapy in treating temporomandibular dysfunction patients. Cranio.

[9] Thumati P., Thumati R.P., Kerstein R.B., Radke J. (2021). Bruxism improvements after disclusion time reduction (DTR)-a pilot study. Adv. Dent. Technol. Tech..

[10] Sutter B.A., Girouard P. (2021). Posture stability and forward head posture before and after disclusion time reduction (DTR). A five-year cohort study. Adv. Dent. Technol. Tech..

[11] Thumati P., Kerstein R., Yiannios N., Radke J., Sutter B. (2018). Changes in the Beck Depression Inventory-II scores of TMD participants after measured occlusal treatment. Adv. Dent. Technol. Tech..

[12] Kerstein R.B., Chapman R., Klein M. (1997). A comparison of ICAGD (immediate complete anterior guidance development) to “mock ICAGD” for symptom reductions in chronic myofascial pain dysfunction patients. Cranio.

[13] Kerstein R.B., Radke J. (2006). The effect disclusion time reduction on maximal clench muscle activity level. Cranio.

[14] Sutter B.A., Thumati P., Thumati R.P., Radke J. (2022). Meniere’s disease patients treated with Disclusion Time Reduction (DTR): A retrospective cohort study of 86 patients (Part 1 of 4). Adv. Dent. Technol. Tech..

[15] Thumati P., Thumati R.P., Kerstein R.B., Radke J. (2022). Trigeminal Neuralgia Improvements After Disclusion Time Reduction (DTR). Adv. Dent. Technol. Tech..

[16] Goud S.S., Thumati P., Poovani S.K., Radke J., Kerstein R.B. (2026). A prospective single arm study of salivary cortisol changes in muscular temporomandibular disorders patients following computer-guided occlusal adjustments. J. Prosthet. Dent..

[17] Podoloff R., Harty M. (2025). T-Scan 10 Recording Dynamics, Force and Timing Software Tools, and the Chairside Clinical Skills for Optimal T-Scan Implementation. Handbook of Research on T-Scan Technology Applications in Dental Medicine (4 Volumes).

[18] Kerstein R.B., Wright N. (1991). An electromyographic and computer analysis of patients suffering from chronic myofascial pain dysfunction syndrome, pre and post-treatment with immediate complete anterior guidance development. J. Prosthet. Dent..

[19] Kerstein R.B. (1994). Disclusion Time Measurement Studies: A comparison of Disclusion Time length of chronic myofascial pain dysfunction syndrome patients and non-patients. A population analysis. J. Prosthet. Dent..

[20] Kerstein R.B. (1994). Disclusion time measurement studies, Stability of disclusion time. A 1-year follow-up study. J. Prosthet. Dent..

[21] Kerstein R.B., Radke J., Sutter B.A., Thumati P., Girouard P. (2026). Canine Protected Occlusion (CPO) Assessed from the Neurological and Muscle Activity Reducing Evidence-based Perspectives. A rebuttal to: Rinchuse; et al., CPO guidance, CRANIO. 2025, 21, 1–8. Adv. Dent. Technol. Tech..

[22] Haines D.E. (2012). Neuroanatomy. An Atlas of Structures, Sections, and Systems.

[23] Kerstein R.B. (2015). Handbook of Research on Computerized Occlusal Analysis Technology Applications in Dental Medicine.

[24] https://www.definitions.net/definition/2013.

[25] Faraguna U., Ferrucci M., Giorgi F.S., Fornai F. (2019). The functional anatomy of the reticular formation. Front. Neuroanat..

[26] Kerstein R.B. (2004). Combining Technologies: A Computerized Occlusal Analysis System Synchronized with a Computerized Electromyography System. Cranio.

